# Interactional skills training in undergraduate medical education: ten principles for guiding future research

**DOI:** 10.1186/s12909-019-1566-2

**Published:** 2019-05-15

**Authors:** Rob Sanson-Fisher, Breanne Hobden, Mariko Carey, Lisa Mackenzie, Lisa Hyde, Jan Shepherd

**Affiliations:** 1 Health Behaviour Research Collaborative, School of Medicine and Public Health, Faculty of Health and Medicine, University of Newcastle, Level 4 West, HRMI Building, Callaghan, NSW 2308 Australia; 2 Priority Research Centre for Health Behaviour, Faculty of Health and Medicine, University of Newcastle, Callaghan, New South Wales 2308 Australia; 3 Hunter Medical Research Institute, New Lambton Heights, New South Wales Australia

**Keywords:** Communication skills, Evidence-based medicine, Undergraduate, Medical education research, Best evidence medical education

## Abstract

**Background:**

High-quality healthcare requires practitioners who have technical competence and communication skills. Medical practitioners need interpersonal skills for gathering and transferring information to their patients, in addition to general consultation skills. Appropriate information gathering increases the likelihood of an accurate diagnosis. Transferring information should be performed in a way that promotes patient understanding and increases the probability of adherence to physician recommendations. This applies to: (i) primary prevention such as smoking cessation; (ii) secondary prevention including preparation for potentially threatening interventions; and (iii) tertiary care, including breaking bad news regarding treatment and prognosis.

**Discussion:**

This debate paper delineates factors associated with undergraduate medical communication skills training where robust research is needed. Ten key principles are presented and discussed, which are intended to guide future research in this field and ensure high quality studies with methodological rigour are conducted.

**Summary:**

The literature on communication skills training for medical school undergraduates continues to grow. A considerable portion of this output is represented by commentaries, descriptive studies or poorly designed interventions. As with any field of healthcare, quality research interventions are required to ensure practice is grounded in high-level evidence.

## Background

Communication, interactional and interpersonal skills are integral to high quality health care [1–6]. Donabedian [1] referred to two complex and intertwined aspects as essential to high-quality care: (1) the technical domain involving decisions about diagnoses and treatment strategies; and (2) the interpersonal domain, considered as the ‘art of medicine’. More recently, there has been a refinement of the concept of interpersonal skills in medical care comprising of three broad domains. The first involves information gathering from the patient required for making an accurate diagnosis [5, 6]. The second involves transferring information back in a way that enables patients to act as partners in their care [6, 7]. To do this, patients must be provided with information that can be understood, retained and applied. This domain includes communication of information on diagnosis and prognosis; the pros and cons of treatment options; and self-management advice about what the patient can do to improve their wellbeing (e.g. management of treatment side effects), prevent future health problems (e.g. smoking cessation advice), or minimize likelihood of treatment complications (e.g. advise about the benefits of early ambulation following surgery). The third domain is often considered to be general interactional skills and covers issues such as opening and closing consultations, controlling the time spent within the consultation, empathy, genuineness and warmth [5, 6].

A seminal paper published in the Lancet in 1980 highlighted the potential benefit of teaching communication skills in the undergraduate medical program [8]. Since this time there has been enormous growth in research investigating this area. While there appears to be much literature in the area, it primarily consists of descriptive or poorly designed intervention research [4, 9]. The importance of evidence based practice is recognised by leading health bodies across the globe, including the World Health Organisation [10], the Institute of Medicine [11] and the National Institute for Health and Care Excellence (NICE) [12]. Within this approach, the need for rigorous research has been emphasised to improve methodological rigor and reduce potential bias when making recommendations for clinical practices [13]. As in any field of health care, continued progression requires high-level evidence to guide its future development. The author’s acknowledge that interactional skills training may be built upon important theoretical aspects, as well as subjective aspects of skills training. It has also been suggested in previous literature that perceptions of good interactional skills will differ between patients, physicians and researchers, increasing the level of complexity of research in this field [14]. Nevertheless, a recent systematic review of studies examining interactional skills training indicate that this field is lacking in methodologically rigorous intervention studies [9] and therefore there is a need to advance this field. This debate paper will focus on providing concrete and specific examples of how research performed in undergraduate skill training could be more methodologically rigorous. It will delineate factors that require robust research and critical thinking if we are to further improve the quality of interactional skills training provided to undergraduate medical students.

## Ten guiding principles for future interactional skills training research

1. Justify interactional skills based on accepted principles

It is important to have clear and explicit principles by which we can determine which interactional skills and domains or topics should be incorporated into undergraduate medical education. Without these principles, undergraduate interactional skill learning outcomes may be based on individual opinion, as well as faculty time and resource availability. Consensus guidelines from the United Kingdom suggest four key principles for governing which interactional skills and topics are incorporated into undergraduate medical training programs: (i) the need for awareness of and familiarity with ethics and the law; (ii) the need for reflective practice to support personal development; (iii) the need for professionalism, including honesty, integrity and strong professional boundaries; and (iv) the need for evidence-based practice [15]. Principle four supports the teaching of skills that have been demonstrated to result in improved patient outcomes. Given that healthcare provision should be driven by level I evidence [4], principle four is particularly important.

Despite the importance of evidence to inform the content of training, there is surprisingly little high quality evidence about the impact of specific interactional skills interventions on patient outcomes [16]. A review of communications skills training for medical students on breaking bad news found only seven relevant studies, of which four were randomized controlled trials. Only one of these studies examined the impact of the training on patient outcomes [17]. Similarly a review on teaching empathy found only four studies examining the impact of interactional skills training on medical students’ empathy, with none of these randomized controlled trials [18]. Well-designed intervention trials are needed to identify interactional skills that are most effective in improving patient outcomes. If such studies are conducted, the findings should inform the interactional skills training content of undergraduate medical education programs.

2. Use methodologically rigorous research to demonstrate that interactional skills can be acquired

Kurtz et al. suggested that individuals cannot be taught interactional skills but rather that these skills are innate [19]. However, undergraduate medical education is predicated on the assumption that a variety of procedural, diagnostic and other skills can be taught, despite some students performing better than others. Evidence that interactional skills can be acquired exists across health care disciplines, including medical, dentistry and nursing education [20]. Roche et al. (1996) performed one of the earlier studies which demonstrated the possible acquisition of interactional skills to enhance smoking cessation advice amongst fifth-year medical students [21]. Those exposed to intervention arms, including audio feedback, role-plays and video feedback, demonstrated significantly improved skills when compared to students experiencing traditional didactic teaching approaches. More recently, similar positive effects of training have been reported in the areas of history taking [22], information retrieval [20] and empathy [23]. However, the volume of research is small, and recent high quality intervention studies are lacking [9]. It is important that continued randomized controlled trials are conducted to ensure that level I evidence is established to show that undergraduate students can acquire interactional skills in areas such as breaking bad news, empathy, information elicitation and provision, and preventive health guidance.

3. Describe training programs in sufficient detail to allow replication

It is essential that health care interventions are described in sufficient detail to allow replication. This allows researchers to build upon the existing evidence base, and for practitioners to implement successful interventions [24]. However, interventions are often poorly described in the peer-reviewed literature. For example, a recent review of interventions for smoking cessation among pregnant women found, of 30 studies meeting basic methodological criteria, not one was deemed as replicable and translatable [25]. These studies included 45 intervention arms, however the training given to providers was not reported in 34 (77%) arms, and similarly, the method of quit advice was not reported in 34 (76%) arms [25]. Training programs for undergraduate medical students should therefore include sufficient information to allow successful replication. Without this, there can be no guarantee that the training will achieve consistent results. While there are a number of papers describing how interactional skills are taught in particular institutions or by clinical researchers, such papers often do not report details such as: how many sessions were involved in the training program; the duration of each session; the role of the tutor; and the specific strategies that were used to increase the probability of acquiring taught skills. Without this level of detail, the training programs being evaluated cannot be replicated. Checklists such as TIDieR (Template for Intervention Description and Replication) [24] may be used to standardize reporting of intervention skills training programs to ensure that all relevant details for replication of training techniques are included within the published literature.

4. Perform assessment in the most robust and reliable manner

The most commonly used, and potentially the least valuable mode of evaluating training effectiveness, is through student questionnaires that typically assess satisfaction or confidence after training. While such surveys are an important way of monitoring quality of undergraduate courses, they do not provide information about the impact of specific training techniques on students’ interactional skills. If assessment methods do not examine the ability of students to demonstrate specific skills, it is unlikely that they will be motivated to attain such skills.

The widespread use of evaluations of satisfaction is particularly disappointing as medical schools typically use a variety of assessment techniques to assess knowledge and skill acquisition in other domains. These include written examinations, assessments from supervising physicians, or direct observation [26]. Direct observation may involve a supervising physician directly observing an interaction with a patient, reviewing a video tape of a consultation, or undertaking a clinical simulation [26]. Clinical simulations typically use actors to simulate patients within specific scenarios known as Objective Structured Clinical Examinations (OSCEs) [26]. The student’s performance is rated against a checklist by the simulated patient or an observer. OSCEs have been developed to assess interactional skills in breaking bad news, disclosing a medical error, handling a disruptive patient, and dealing with a phone call for a narcotics refill [27]. The key advantage of simulations is that they enable students to demonstrate the application of knowledge and skills to a clinical scenario. Methodological limitations, however, include reactivity whereby students may perform differently under exam conditions compared to how they would in day-to-day clinical interactions with patients. One potential way of eliminating the impact of reactivity is the use of simulated patients to assess skills where the student is unaware that the patient is “simulated”. While not commonly used in medical education, this type of assessment may provide a better indication of the extent to which the student is able to apply skills in real world contexts.

5. Evaluate cost effectiveness of training programs

Information about both cost of delivering communication skills training and the impact of such training on student skills and other outcomes is needed to determine cost effectiveness [28]. This information is essential to determining the value for money in medical education [28]. Communication skills training typically involves a combination of didactic and interactive teaching, including tutorials involving small groups of students role playing the skills they have learned [29]. Simulated patients may be used in the role plays, and feedback is typically considered a core part of communication skills training [29]. This may be considered a labour intensive form of teaching. Additionally, there is competition for space in the undergraduate medical curriculum; therefore, it is important to determine how much time should be devoted to this in order to ensure that students obtain the required skills. Despite this, few papers report on the costs or cost effectiveness of delivering communication skills training and assessing student skills. Kelly and Murphy (2004) completed one study evaluating the cost of designing, delivering and assessing medical students’ communication skills using OSCE [30]. They reported that personnel costs far exceeded that of standardized patient costs and administrative costs, and that OSCE was expensive to run. Given continuing economic pressures in medical education, and that Kelly and Murphy’s study was completed over a decade ago, further evaluations are needed to establish basic cost effectiveness information. This may include evaluating the cost of different teaching and assessment approaches, such as how the format, content, duration and intensity of training can be altered to maximize cost effectiveness.

6. Provide evidence concerning the characteristics of the most successful teachers

Consideration of which discipline offers the most effective teachers for interaction skills training may be important for maximising student outcomes. Teachers of communication skills in undergraduate medical programs may be from disciplines where communication is considered a central skill, such as behavioral science, psychology, psychiatry or family medicine. Teachers from these disciplines may have an in-depth knowledge of theory and evidence underpinning communication strategies, but may not have experience in the topic at hand. For example, a psychologist is not likely to have had experience explaining treatment options and obtaining informed consent for medical treatment. Alternatively, teachers may be medical specialists who have experience relevant to the content area. For example, medical oncologists or surgeons could teach how to deliver the bad news of a cancer diagnosis. A possible advantage of medical specialists training students is that they can draw on their practical experience in applying evidence-based strategies. Medical specialists may also have an important role to play in formal teaching and informal (in the clinic) role modelling of communication skills [31]. Good role models require a combination of clinical skills, teaching skills and personal qualities such as honesty and compassion [31]. Having a respected professional demonstrate a commitment to using communication skills, both verbally and behaviorally, may serve as a motivator for students to learn and adopt the skills taught. One review based on descriptive and commentary papers identified interpersonal factors such as ability to actively engage with students, to communicate well and inspire students as important teacher characteristics [32]. However, there is a lack of experimental evidence on this topic [32]. Furthermore, there are no experimental studies examining whether the discipline of the interactional skills teacher has an impact on student outcomes. Hence, such experimental work should be the subject of subsequent research.

7. Consider timing of specific interactional skills training within the undergraduate course

The timing of interactional skills training in the undergraduate medical course may influence how these skills are applied in clinical practice. Descriptive research indicates that integration of skills development throughout the early stages of the curriculum assists with students’ perceived preparation for their clinical practice training [33], and that longitudinal, integrated training (rather than intensive, concentrated training) leads to higher level communication skills [34]. To the best of our knowledge, there are no published recommendations on when specific interactional skills should be taught to undergraduates to ensure long-term competency and translation into improved patient outcomes. It may be expected that basic interactional skills (e.g. communication skills for information gathering) [35] will be taught in the earlier years of the undergraduate program, and more complex topics, such as breaking bad news, taught in the later years [36–38]. Early introduction of skills training may also lead to increased opportunities for repetitive practice [39], and for obtaining specific feedback on the quality of their interactional skills [40], if students have a learning agenda to prioritize this in problem-based learning scenarios or clinical environments [20, 41, 42]. In the absence of evidence-based recommendations for course content and timing, it is likely that pragmatic considerations have guided the timing of interactional skills training within crowded undergraduate medical program curricula [37, 43]. Evaluating the effectiveness of training at different stages of the undergraduate program could guide decisions about the timing of communication skills training within undergraduate medical curriculum.

8. Focus on training in both information gathering and information transfer skills

Undergraduate medical students are expected to develop competency in information transferring as well as information gathering skills. Though information gathering and acquiring medical histories has long been part of medical training, less emphasis has been placed on information transfer [38]. This is despite skills in eliciting information only making up a small subset of the range of core communication skills deemed by health professional education bodies internationally to be key aspects of training for medical and other health professionals [44]. While the interaction between patients’ and providers’ is collaborative and relational, emerging literature reporting on the quality of patient-centered communication and care seems to consistently highlight that many clinicians have some deficiency in the communication skills required for effective information transfer [5]. It would, therefore, appear that undergraduate communication skills training should be weighted towards ensuring training and practice in these skills. For example, in a sample of 268 haematology cancer patients, 44% reported that they were not involved in treatment decision making in accordance with their preferences [45], potentially leading to poor adherence to treatment. There is a need for more controlled research into effective training approaches for transferring potentially sensitive or complex information in a patient-centered manner.

9. Evaluate whether interactional skills generalize over time

Transfer of knowledge is said to have been successful when a learned skill is generalized into clinical practice, maintained over time and applied across settings [46]. For example, a student might be taught interactional skills in the final year of their undergraduate course with the expectation that those skills would be used throughout internships, registrar training and consulting years. However, those studies which do exist in the field suggest that these skills degrade over time even when there is an explicit attempt to assess the student [47]. In some ways, this is not unexpected. Training during the undergraduate program is able to demonstrate that an individual can acquire the skills but whether or not they generalize over time is a function of many variables. This might include whether the skills have been taught, demonstrated and reinforced by peers and senior colleagues in the clinical environment; if the skills are observed, monitored and corrected by those responsible for postgraduate training; and whether systems are in place to provide feedback on whether key standards are met and potential contingencies if they are not achieved. Roche et al. demonstrated that while interns may be asked to watch a clinician perform technical aspects of care, and then demonstrate that competency and receive feedback, the same could not be said for important interaction skills such as breaking bad news, or modification strategies to reduce harmful behaviors such as alcohol and tobacco use [48]. Furthermore, a recent systematic review examining teaching communicating bad news to surgery residents highlighted that the research in this field is lacking and that there are several impediments to teaching these skills beyond undergraduate courses [49]. There is a need to find mechanisms by which interactional skills can be monitored over time and strategies to increase the probability that generalisation of these skills occur.

10. Evaluate whether taught skills transfer across clinical specialties

In addition to generalising skills over time, designated interactional skills should be applied across clinical settings. Technical skills inevitably differ between medical specialties; however, interactional skills are important and relevant across all specialties. Nevertheless, previous research has suggested that interactional skills are lacking in surgical trainees [50, 51] and patient-centeredness differs between medical specialties [52]. While medical students may understand that general interactional skills, such as empathy and warmth, are important for all practicing doctors, they may also perceive that certain skills are more relevant to some specialties compared to others. However, when taking the example of preparing patients for potentially threatening procedures, this is of obvious importance to surgeons but is also important for oncologists preparing patients for cancer treatment and general practitioners preparing patients for minor procedures such as needles or pap smears. Similar examples could be applied for delivering preventative health care and breaking bad news. If competency of interactional skills is determined by clinical specialties, it is likely that experiences among these specialties may shape medical students’ ongoing behaviors, thus perpetuating sub-optimal communication among specialty areas. Further research is needed to ensure that interactional skills are implemented and evaluated within different settings and that medical students are trained in the importance of transferring these skills across specialties.

## Conclusions

This debate paper argues for 10 principles which could be used to guide future research and acquire the evidence base for training undergraduate medical students in communication skills. We acknowledge that there are considerable pragmatic and experimental problems in acquiring this information. There is an obligation, however, for those working in the field of interactional skills to ensure research efforts are grounded in methodologically sound data. Not performing high quality research for interaction skills training decreases the probability of improving the quality of care provided to patients within the health care system.

## Abbreviations

NICE: National Institute for Health and Care Excellence
OSCEs: Objective Structured Clinical Examinations
TIDieR: Template for Intervention Description and Replication
