# Correction to: Interactional skills training in undergraduate medical education: ten principles for guiding future research

**DOI:** 10.1186/s12909-019-1695-7

**Published:** 2019-07-23

**Authors:** Rob Sanson-Fisher, Breanne Hobden, Mariko Carey, Lisa Mackenzie, Lisa Hyde, Jan Shepherd

**Affiliations:** 1 Health Behaviour Research Collaborative, School of Medicine and Public Health, Faculty of Health and Medicine, University of Newcastle, Level 4 West, HRMI Building, Callaghan, NSW 2308 Australia; 2 Priority Research Centre for Health Behaviour, Faculty of Health and Medicine, University of Newcastle, Callaghan, NSW 2308 Australia; 3 Hunter Medical Research Institute, New Lambton Heights, NSW Australia

**Correction to: BMC Med Educ**

**https://doi.org/10.1186/s12909-019-1566-2**

Following publication of the original article [1], the authors reported a referencing error under the heading 2. Use methodologically rigorous research to demonstrate that interactional skills can be acquired in the Background section of the published article.

**Incorrect referencing in published article:**

Kurtz et al. suggested that individuals cannot be taught interactional skills but rather that these skills are innate [19].

**Corrected referencing:**

It has been suggested that individuals cannot be taught interactional skills but rather that these skills are innate.
